# Chylous ascites and chylothorax due to constrictive pericarditis in a patient infected with HIV: a case report

**DOI:** 10.1186/1752-1947-6-163

**Published:** 2012-06-27

**Authors:** Sarawut Summachiwakij, Wiwun Tungsubutra, Pornpan Koomanachai, Suchai Charoenratanakul

**Affiliations:** 1 Department of Physiology, Faculty of Medicine Siriraj Hospital, Mahidol University, Bangkok, 10700, Thailand; 2 Department of Medicine, Faculty of Medicine Siriraj Hospital, Mahidol University, Bangkok, 10700, Thailand

## Abstract

**Introduction:**

Chylothorax and chylous ascites are uncommon and usually associated with trauma or neoplasms. To the best of our knowledge, constrictive pericarditis leading to chylothorax and chylous ascites in a person infected with HIV has never previously been described.

**Case presentation:**

A 39-year-old Thai man was referred to our institute with progressive dyspnea, edema and abdominal distension. His medical history included HIV infection and pulmonary tuberculosis that was complicated by tuberculous pericarditis and cardiac tamponade. Upon further investigation, we found constrictive pericarditis, chylothorax and chylous ascites. A pericardiectomy was performed which resulted in gradual resolution of the ascites and chylous effusion.

**Conclusions:**

Although constrictive pericarditis is an exceptionally rare cause of chylothorax and chylous ascites, it should nonetheless be considered in the differential diagnosis as a potentially reversible cause.

## Introduction

Chylothorax and chylous ascites are usually associated with trauma or neoplasms, particularly lymphomas. Constrictive pericarditis is an extremely rare cause of chylothorax and chylous ascites. In the English literature, there are only three case reports of both chylothorax and chylous ascites due to constrictive pericarditis [1-3]. In these three case reports, none of the patients had HIV infection. There is only one case report of a patient infected with HIV with chylothorax and chylous ascites and the etiology was tuberculosis-associated immune reconstitution inflammatory syndrome (TB-IRIS) [4]. To the best of our knowledge, constrictive pericarditis leading to chylothorax and chylous ascites in patients infected with HIV has never been previously described.

## Case presentation

A 39-year-old Thai man was referred to our institute for a pericardiectomy. He initially presented with progressive exertional dyspnea, increased abdominal girth and leg edema for nine months. He denied having cough, fever, chills, night sweats or weight loss. He was diagnosed as having HIV infection and pulmonary tuberculosis six years previously, which was complicated by pericarditis and cardiac tamponade. He was subsequently treated with antituberculosis therapy, which resulted in a cure, and he was started on a combination of stavudine, lamivudine and nevirapine.

On physical examination, he was afebrile with the presence of marked edema in the lower extremities. His jugular veins were distended up to the mandible. He was found to have decreased breath sounds in the left lower lung area. The results of an abdominal examination were remarkable for ascites as evidenced by shifting dullness.

**Figure 1:**
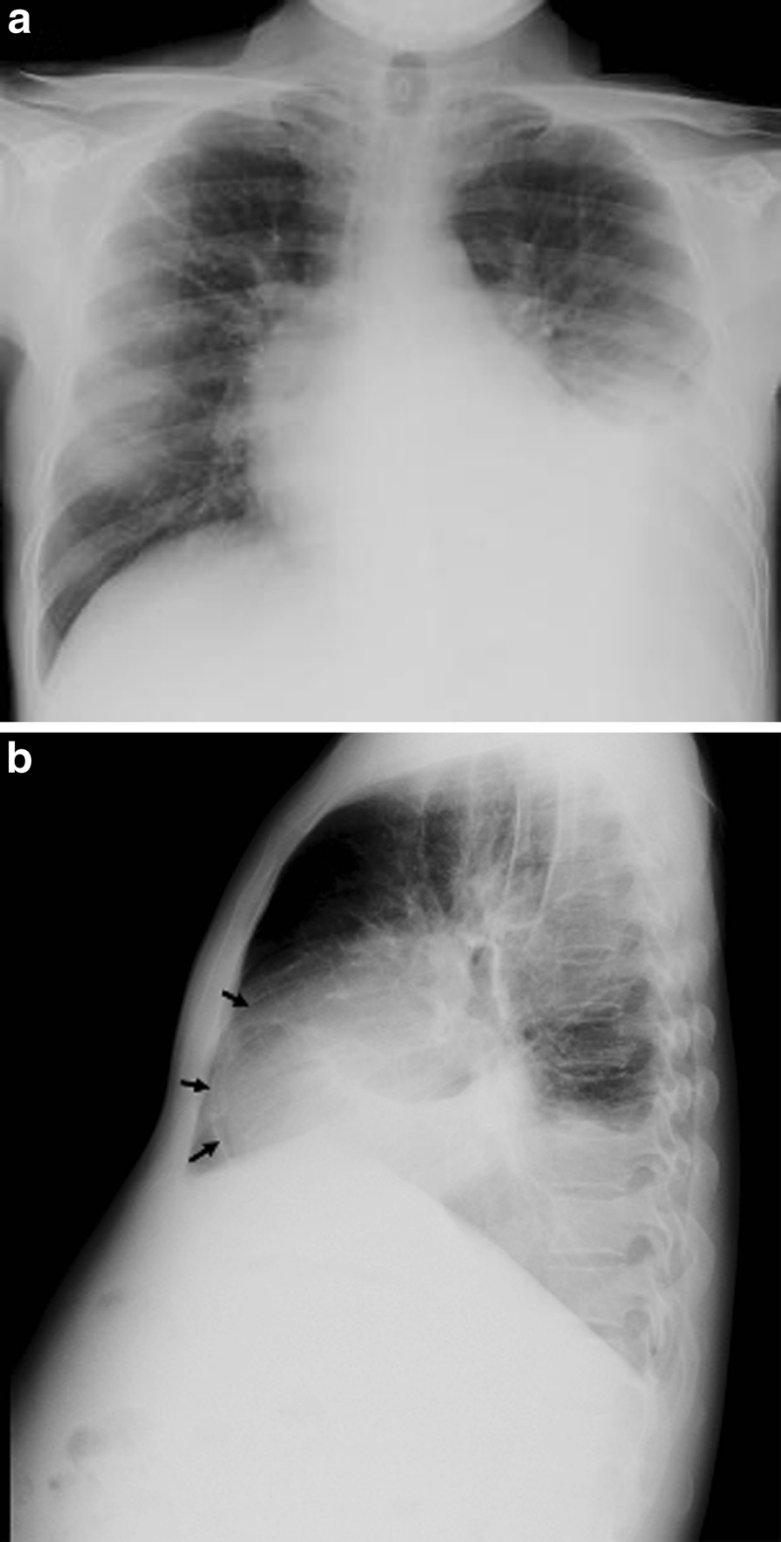
**Radiograph of the chest.** Posterior anterior view **(A)** showing left-sided pleural effusion. Lateral view **(B)** showing pericardial calcification (arrows).

The results of a sputum examination were negative for *Mycobacterium tuberculosis.* The chest radiograph revealed minimal fibroreticular infiltration in the right upper lung, a left-sided pleural effusion and a calcified pericardium (Figure 1A,B). Transthoracic echocardiography demonstrated normal left ventricular size with preserved left ventricular systolic function and bi-atrial enlargement. The Doppler findings were consistent with the diagnosis of constrictive pericarditis.

**Figure 2:**
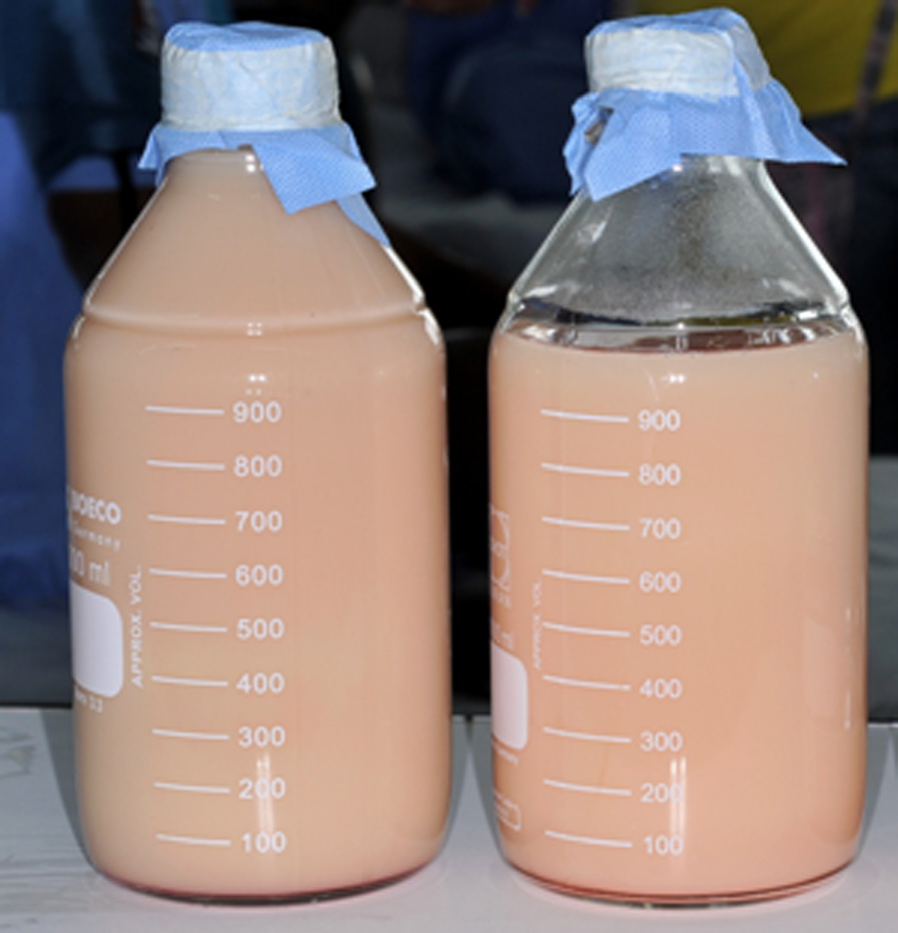
Bottles containing milky fluid drained from left pleural cavity.

Thoracocentesis yielded a milky fluid (Figure 2) with a total protein level of 1.6g/dL, albumin 0.9g/dL, glucose 130mg/dL, lactate dehydrogenase (LDH) 128U/L, triglyceride 475mg/dL, cholesterol 39mg/dL and an adenosine deaminase (ADA) level of 40.6U/L (normal <45U/L). The cell count of the pleural fluid was 78 cells/mm^3^ with 55% lymphocytes and 29% neutrophils. Abdominal paracentesis also revealed a milky fluid and showed a total protein level of 1.7g/dL, albumin 0.9g/dL, triglyceride 535mg/dL and a cell count of 160 cells/mm^3^ with 79% lymphocytes and 16% neutrophils. All tests for *M. tuberculosis*, bacteria, and fungi from the pleural and peritoneal fluids and blood cultures were negative. In addition, a cytological examination was negative for malignant cells.

**Figure 3:**
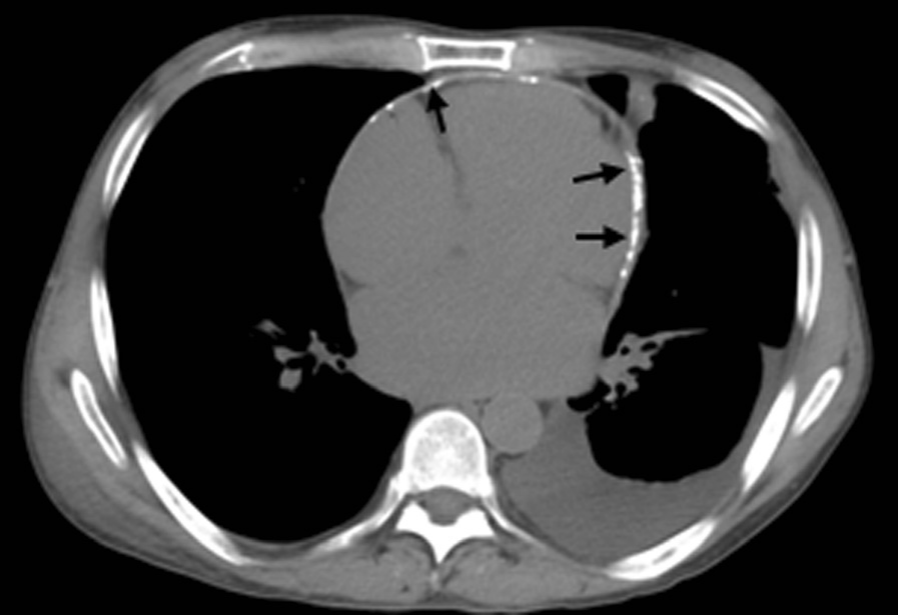
Computed tomography (CT; axial view) of the chest showing left-sided pleural effusion and a calcified pericardium (arrows).

Computed tomography (CT) of the chest showed a left-sided pleural effusion and multiple lymphadenopathies at the right hilar, right paratracheal and precarinal region (Figure 3). An abdominal CT scan showed multiple small intra-abdominal lymphadenopathies. A left adrenal gland mass 3.4×1.5cm in size was noted, and a small nodule at the right adrenal gland was also detected. A percutaneous CT-guided biopsy of the left adrenal mass was performed. A histopathological examination revealed a granulomatous inflammation containing a monomorphic yeast-like organism, which was morphologically suggestive of a *Histoplasma* species.

A bone marrow aspiration demonstrated no tumor or granuloma; cultures for bacteria, fungi and mycobacteria were negative.

A lymphoscintigraphy with combined single-photon emission computed tomography/CT (SPECT/CT) imaging revealed an absence of radioactive accumulation within the pleural or peritoneal cavity in the early phase, thus excluding significant leaks from the main lymphatic tracts. On delayed images, there was radioactive accumulation in the abdomen that suggested an increased abdominal lymphatic production and a restriction of lymphatic drainage at the left subclavian vein.

Our patient underwent a pericardiectomy and intra-operative findings demonstrated a calcified, thickened pericardium with adhesion to the heart. Histopathological findings showed pericardial fibrosis with focal calcification and no granulomas. The mediastinal lymph nodes showed a reactive change and pericardial tissue cultures for bacteria, fungi, and mycobacteria were negative.

He was given a high-protein, low-fat diet with medium-chain triglycerides, and amphotericin B was administered to treat the disseminated histoplasmosis with adrenal involvement. Our patient was discharged home one month after the operation with resolution of his ascites and pleural effusion.

## Discussion

Chylothorax and chylous ascites due to constrictive pericarditis is a very rare situation. In the English literature, there have only been three cases of constrictive pericarditis with chylothorax and chylous ascites [1-3]. Chylothorax and chylous ascites are caused by accumulation of chyle in the pleural and peritoneal space. A diagnosis of chylothorax and chylous ascites is made when the fluid triglyceride levels are more than 110mg/dL [5]. These conditions are usually produced by obstruction and disruption of the thoracic duct or one of its major divisions, which usually is the result of malignancy, trauma or inflammation [6]. Other conditions that have been reported to be the cause of chylothorax and chylous ascites, include liver cirrhosis, hypothyroidism, nephrotic syndrome and abdominal operations [7]. Notwithstanding the above conditions, the coexistence of chylothorax and chylous ascites also can be caused by cardiac problems, such as ischemic cardiomyopathy, constrictive pericarditis and cardiac amyloidosis [8].

With our patient, an abdominal and thoracic CT, as well as cytology of the pleural fluid and ascites, were performed to exclude any malignancies. The adrenal histoplasmosis was an incidental finding. Studies of the bone marrow and the mediastinal lymph nodes did not reveal any evidence of malignancy or infection. The assumption that chylothorax and chylous ascites occurred as a result of constrictive pericarditis is supported by the progressive resolution of the pleural effusion and ascites after pericardiectomy.

Chylous ascites resulting from constrictive pericarditis could potentially be produced by the following mechanisms. An elevated central venous pressure increases capillary infiltration, which leads to increased lymphatic production whereby the lymph flow of the thoracic duct rises. However, the stiffness of the venolymphatic junction limits the return of the increased lymphatic flow to the systemic circulation [9]. Moreover, the high pressure in the left subclavian vein may further reduce the lymphatic drainage [6]. The lymphatic venous collaterals cannot develop effectively in the presence of increased venous pressure and, consequently, the increased lymphatic pressure leads to the rupture of dilated intestinal lacteals and lymphatic leakage into the peritoneal cavity [10]. Moreover, chylous ascites could probably pass from the peritoneal to the pleural cavity through small diaphragmatic defects, similar to the mechanisms of hepatic hydrothorax [11].

The summaries of the three previous cases of constrictive pericarditis reported to be associated with both chylothorax and chylous ascites are detailed in Table 1. The three patients, and our patient’s case, had resolution of the chylous effusion and ascites after pericardiectomy. The second patient had persistent chylothorax that subsequently resolved after the administration of octreotide [2]. The etiology of constrictive pericarditis in the first case was not established [1]. In this case, the patient had chylothorax and subsequently developed chylous ascites following a thoracic band ligation. It is uncertain whether the cause of chylous ascites was thoracic duct ligation or constrictive pericarditis. In the second case, it was due to chronic resolving hemopericardium associated with repeated chest trauma from boxing [2]. In the third case, it might have resulted from uremic or dialysis pericarditis [3].

**Table 1:** Cases with chylothorax and chylous ascites resulting from constrictive pericarditis in the medical literature

**Case no.**	**Year**	**Age (years)/sex**	**Medical history**	**Etiology of constrictive pericarditis**	**Treatment**	**Result**	**Reference**
1	2003	35/M	Aortoplasty for aortic coarctation at five years old	Not known	Pericardiectomy	Recovery	[1]
2	2003	65/M	Hypertension	Repeated chest trauma	Pericardiectomy, octreotide	Recovery	[2]
3	2004	41/M	Chronic renal failure	Uremic or dialysis pericarditis	Pericardiectomy	Recovery	[3]
4	2011	39/M	HIV	Tuberculous pericarditis	Pericardiectomy, medium-chain triglyceride	Recovery	Present case

In our patient’s case, we believe the etiology of constrictive pericarditis was most likely due to tuberculosis pericarditis since he had a history of tuberculous pericarditis. Moreover, the histopathology characterized by scarring and calcification is consistent with the constrictive phase of tuberculous pericarditis [12]. Histoplasmosis has been reported to cause constrictive pericarditis [13]. Furthermore, mediastinal granulomatous disease caused by *H. capsulatum* infection may lead to mediastinal fibrosis as a result of excessive fibrogenic response to the antigen of the fungus. Fibrosing mediastinitis can cause several mediastinal complications, including constrictive pericarditis [14]. In our patient’s case, the pericardial tissue and mediastinal lymph nodes did not reveal evidence of histoplasmosis. In addition, the chest CT scan results were not suggestive of fibrosing mediastinitis, which is characterized by mediastinal or hilar mass, calcified lymph nodes and tracheobronchial narrowing [15]. Regardless, the adrenal histoplasmosis indicated a disseminated form; thus, the patient received amphotericin B to treat the disseminated histoplasmosis.

To the best of our knowledge, the case of a patient infected with HIV with both chylothorax and chylous ascites due to constrictive pericarditis has never been previously described. Chylous ascites in patients infected with HIV is rare and commonly due to infections and immune reconstitution inflammatory syndrome (IRIS). Only one patient infected with HIV has been reported to have both chylothorax and chylous ascites associated with TB-IRIS [4].

## Conclusions

We describe a case of a patient infected with HIV with constrictive pericarditis who was found to have chylothorax and chylous ascites. Tuberculous pericarditis was the most likely etiology of the constrictive pericarditis. He underwent a pericardiectomy with subsequent resolution of the chylothorax and chylous ascites. To the best of our knowledge, there is no other case report on the combination of chylothorax and chylous ascites due to constrictive pericarditis in patients infected with HIV.

## Consent

Written informed consent was obtained from the patient for publication of this case report and any accompanying images. A copy of the written consent is available for review by the Editor-in-Chief of this journal.

## Competing interests

The authors declare that they have no competing interests.

## Authors’ contributions

SS performed the literature review and was a major contributor in writing the manuscript. WT contributed to patient management, gathered patient information, and wrote and edited the manuscript. PK contributed to patient management, performed the literature review and edited the manuscript. SC contributed to patient management and edited the manuscript. All authors have read and approved the final manuscript.
